# National

**Published:** 1896-08

**Authors:** 


*National.* Secretary Morton has declined to raise the quarantine regulations against cattle from France, Swizerland, and Belgium.

The presence of foot and mouth, as well as other diseases, demands protection for our cattle-owners, and now that we have been free from contagious pleuro-pneumonia for three years or more, we should not risk again implanting it upon our soil.

